# Comparison of four outdoor pilot-scale photobioreactors

**DOI:** 10.1186/s13068-015-0400-2

**Published:** 2015-12-18

**Authors:** Jeroen H. de Vree, Rouke Bosma, Marcel Janssen, Maria J. Barbosa, René H. Wijffels

**Affiliations:** Bioprocess Engineering, AlgaePARC, Wageningen University, P.O. Box 16, 6700 AA Wageningen, The Netherlands; Food and Biobased Research, AlgaePARC, Wageningen UR, P.O. Box 17, 6700 AA Wageningen, The Netherlands; Biosciences and Aquaculture, Nordland University, 8049 Bodø, Norway; Bioprocess Engineering, AlgaePARC, Wageningen University, P.O. Box 8129, 6721 NG Bennekom, The Netherlands

**Keywords:** Microalgae, Outdoor, Pilot-scale, Photobioreactors, Areal productivity, Photosynthetic efficiency, *Nannochloropsis* sp.

## Abstract

**Background:**

Microalgae are a potential source of sustainable commodities of fuels, chemicals and food and feed additives. The current high production costs, as a result of the low areal productivities, limit the application of microalgae in industry. A first step is determining how the different production system designs relate to each other under identical climate conditions. The productivity and photosynthetic efficiency of *Nannochloropsis* sp. CCAP 211/78 cultivated in four different outdoor continuously operated pilot-scale photobioreactors under the same climatological conditions were compared. The optimal dilution rate was determined for each photobioreactor by operation of the different photobioreactors at different dilution rates.

**Results:**

In vertical photobioreactors, higher areal productivities and photosynthetic efficiencies, 19–24 g m^−2^ day^−1^ and 2.4–4.2 %, respectively, were found in comparison to the horizontal systems; 12–15 g m^−2^ day^−1^ and 1.5–1.8 %. The higher ground areal productivity in the vertical systems could be explained by light dilution in combination with a higher light capture. In the raceway pond low productivities were obtained, due to the long optical path in this system. Areal productivities in all systems increased with increasing photon flux densities up to a photon flux density of 30 mol m^−2^ day^−1^. Photosynthetic efficiencies remained constant in all systems with increasing photon flux densities. The highest photosynthetic efficiencies obtained were; 4.2 % for the vertical tubular photobioreactor, 3.8 % for the flat panel reactor, 1.8 % for the horizontal tubular reactor, and 1.5 % for the open raceway pond.

**Conclusions:**

Vertical photobioreactors resulted in higher areal productivities than horizontal photobioreactors because of the lower incident photon flux densities on the reactor surface. The flat panel photobioreactor resulted, among the vertical photobioreactors studied, in the highest average photosynthetic efficiency, areal and volumetric productivities due to the short optical path. Photobioreactor light interception should be further optimized to maximize ground areal productivity and photosynthetic efficiency.

## Background

Microalgae are a promising feedstock for bulk commodities like chemicals, food, feed and fuels. High production costs hinder the current implementation of algal biomass as a feedstock for bulk commodities; production costs should decrease to less than 1 €/kg dry weight [1]. A crucial parameter influencing biomass production costs is photosynthetic efficiency; the efficiency at which solar light energy is captured as chemical energy in biomass. Under identical conditions, a higher photosynthetic efficiency means a higher ground areal productivity, and thus a decrease in biomass production costs [1, 2].

Microalgae are produced in a wide variety of cultivation systems including open raceway ponds, tubular, and flat panel photobioreactors. Open raceway ponds are ring-channel systems, with a typical depth of 0.2 m. The culture is typically mixed at 0.25 m s^−1^ by a paddle wheel. Open raceway ponds are characterized by low cell densities up to 0.3 g L^−1^ [3]. The open raceway pond is currently the mostly used and cheapest cultivation system for commercial production of microalgae [4]. Norsker et al. estimated an investment cost of 0.37 M€/ha for a 100 ha scale open raceway pond plant [3].

Tubular photobioreactors are made of transparent tubing through which the culture is circulated at liquid velocities of typically 0.5 m s^−1^ [3]. To prevent high oxygen concentrations the transparent tubes are connected to a degasser or stripper vessel, where oxygen is removed by air injection. Tubular systems can be found in different orientations; horizontal tubes arranged in a single plane and multiple planes of vertically stacked horizontal tubes (fence-like systems). Diameters of the tubes vary with system orientation, diameters larger than 3 cm and smaller than 10 cm are typically used [4]. Tubular photobioreactors are more expensive to construct than open raceway ponds, especially vertically oriented tubular photobioreactors. Investment costs for a 100 ha horizontal tubular plant were estimated to be 0.51 M€/ha by Norsker et al. [3].

Flat panel photobioreactors are transparent flat vessels, where the culture is mixed by aeration (≤1 L^−1^ L^−1^ min^−1^ or 1 vvm). The culture depth or optical path in flat panel systems varies from 1 to 20 cm and, consequently, biomass concentrations in these systems vary greatly [5]. For a 100 ha production plant using flat panel photobioreactors (optical path 3 cm), investment costs were estimated to be 0.8 M€/ha [3].

For the selection of a photobioreactor for large scale production, knowledge on the actual productivity and photosynthetic efficiency of different photobioreactor designs is required. Norsker et al. reported an overview of photosynthetic efficiencies obtained with different reactors, locations and microalgal species; 1.5 % for open raceway pond, 3 % for horizontal tubular photobioreactors and 5 % for flat panel photobioreactors [3]. However, for a better comparison of photobioreactor designs data should be gathered at a single location with the same microalgal species. In this study, we simultaneously compared the performance of four pilot-scale outdoor photobioreactors with *Nannochloropsis* sp. under identical climatological conditions in The Netherlands. Four photobioreactors were installed at the AlgaePARC pilot facility; an open raceway pond (OPR), a horizontal tubular photobioreactor (HT), a vertical tubular photobioreactor (VT), and a flat panel photobioreactor (FP) [6]. The effect of daily dilution rates and photon flux densities on areal productivity and photosynthetic efficiency was evaluated for each cultivation system.

## Results and discussion

Areal productivity and photosynthetic efficiency of four different outdoor photobioreactors operated at different dilution rates were determined. The effect of the photon flux density on the areal productivity for each dilution rate is evaluated. Furthermore, the effect of photon flux density on photosynthetic efficiency is evaluated for all systems studied. The horizontal tubular photobioreactor, vertical tubular photobioreactor, open raceway pond and flat panel were in operation for 111, 102, 42 and 77 days, respectively. During these periods, all four systems were restarted three times due to different reasons. In the tubular photobioreactors this was due to fouling, in the OPR this was due to contamination and growth limiting temperatures (<20 °C). The flat panel was restarted because of clogging of aeration holes, resulting in suboptimal operation.

### Effect of photon flux density on productivity

**Fig. 1:**
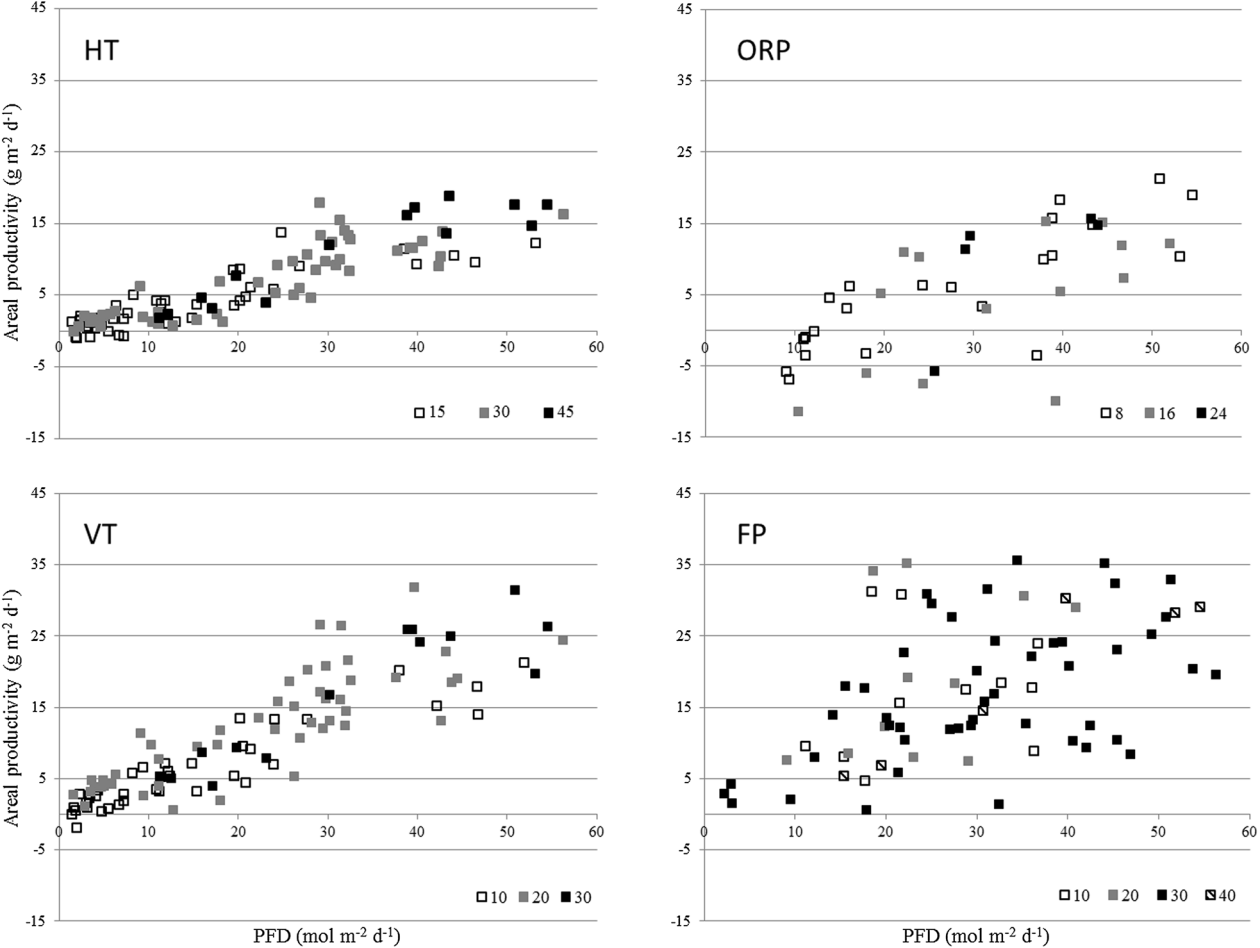
Influence of daily photon flux density and dilution rate on areal productivity. For the horizontal tubular (HT), open raceway pond (ORP), vertical tubular (VT) and flat panel (FP). The *different colors* of markers indicate the different dilution daily rates

In Fig. 1, areal productivities versus photon flux densities are shown for all cultivation systems operated. For all systems, areal productivities increased with higher photon flux densities, indicating cultures could experience light limitation at low photon flux densities. For HT, VT and ORP areal productivities appear to increase linearly with PFD up to 30 mol m^−2^ day^−1^, this has been reported previously by [7–10]. For the flat panel photobioreactor this trend could not be observed, a possible explanation could come from the limited mixing of the culture over the entire reactor. Maximal areal productivities for all systems were obtained above 30 mol m^−2^ day^−1^. Highest areal productivities were obtained with the flat panel photobioreactor. In the vertical tubular system similar areal productivities as in the flat panel photobioreactor were obtained, followed by the horizontal tubular photobioreactor and the raceway pond. The areal productivities in the ORP were low in comparison to the other systems. The large optical path (0.2 m) and long light dark cycles as a result of poor mixing in this system contribute to lower areal productivity [11–13].

In vertical photobioreactors, microalgal cell dissipate less of the absorbed light energy as a result of lower photon flux densities because of light dilution on the reactor surface in comparison to the horizontal systems. Therefore, higher areal productivities were found in vertical systems. The higher photon flux density on the exposed reactor surface of the horizontally oriented cultivation systems results thus in lower areal productivity and photosynthetic efficiency.

The short optical path of the flat panel photobioreactor results in small dark zone in the culture; respiration takes place in a small part of the culture. The long optical path in the open raceway pond results in a large dark zone in the culture. [11–13]. In the dark zone microalgae respire energy that otherwise could be used for growth. The presence of a dark zone in a cultivation system will reduce the net productivity of the culture, as part of the culture in the dark has negative growth. A long optical path results in lower productivities (Fig. 1) [12, 14]. Higher photon flux densities will penetrate deeper in the culture and will decrease the size of the dark zone present in the culture.

Variations in areal productivity were larger for all photon flux densities and dilution rates in the flat panel photobioreactor and the open raceway pond than in the tubular systems. The large variations in the flat panel photobioreactor are a result of the plug flow regime moving the culture through each panel. The culture is not mixed well over all panels, while in the other systems the entire culture volume is mixed resulting in less variation in areal productivity.

In the open raceway pond the large variation in areal productivity is the result of low culture temperatures and automated level control. The low culture temperatures resulted in suboptimal conditions during a large part of the day, for many days throughout the experimental period. The automated level control in the open raceway pond resulted in negative areal productivity; for days with heavy rainfall, dilution rates were higher than intended because of the automated level control.

**Fig. 2:**
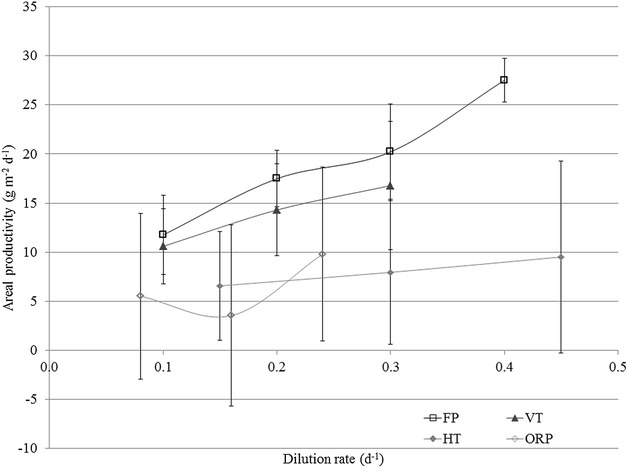
Average areal productivity versus dilution rate for each photobioreactor. Flat panel (FP), vertical tubular (VT), horizontal tubular (HT) and open raceway pond (ORP). Average areal productivity was calculated over a number of days for each dilution rate; FP; 6, 3, 40, 4, VT; 18, 39, and 14, HT; 19, 38, and 11, ORP; 22, 13, and 5

The variations in areal productivities within the different photobioreactors are a result of variations in biomass concentrations. The biomass concentrations in the different cultivation systems varied as a result of applied dilution rates and photon flux densities. The highest dilution rates in the flat panel photobioreactor (0.4 day^−1^) and open raceway pond (0.24 day^−1^) were only applied for a short period of time, 6 and 11 days, respectively, as these resulted in a strong decrease in biomass concentration. For each cultivation system the average areal productivity was calculated for each dilution rate. These average values were calculated over the summer period to ensure similar values for photon flux density (Fig. 2).

The system with the shortest optical path (0.02 m), the flat panel photobioreactor, resulted in the overall highest average areal productivity. The overall lowest average areal productivity was obtained with the open raceway pond, because of the long optical path (0.2 m). The vertical tubular photobioreactor resulted in higher average areal productivity compared to the horizontal tubular. The vertical tubular photobioreactor has a lower photon flux density on the surface of the reactor that penetrates less far in the culture. The horizontal tubular photobioreactor resulted in a higher biomass concentration, as this system receives higher photon flux density. No significant difference in average areal productivity at different dilution rates was found among all cultivation systems with the exception of the flat panel.

### Effect of photon flux density on photosynthetic efficiency

Photosynthetic efficiency (PE_sunlight_) is an important parameter for the evaluation of photobioreactor performance. Photosynthetic efficiency is the efficiency at which solar light energy is captured as stored chemical energy in biomass and it allows the estimation of the productivity for other locations if the photon flux density is known. Photosynthetic efficiency was calculated based on the ground areal productivity and ground areal irradiance. At the same ground areal photon flux density, vertical photobioreactors have lower photon flux densities on the surface of the cultivation system than horizontal systems. Lower photon flux densities result in less energy dissipation by microalgal cells in the form of heat, resulting in a higher photosynthetic efficiency.

**Fig. 3:**
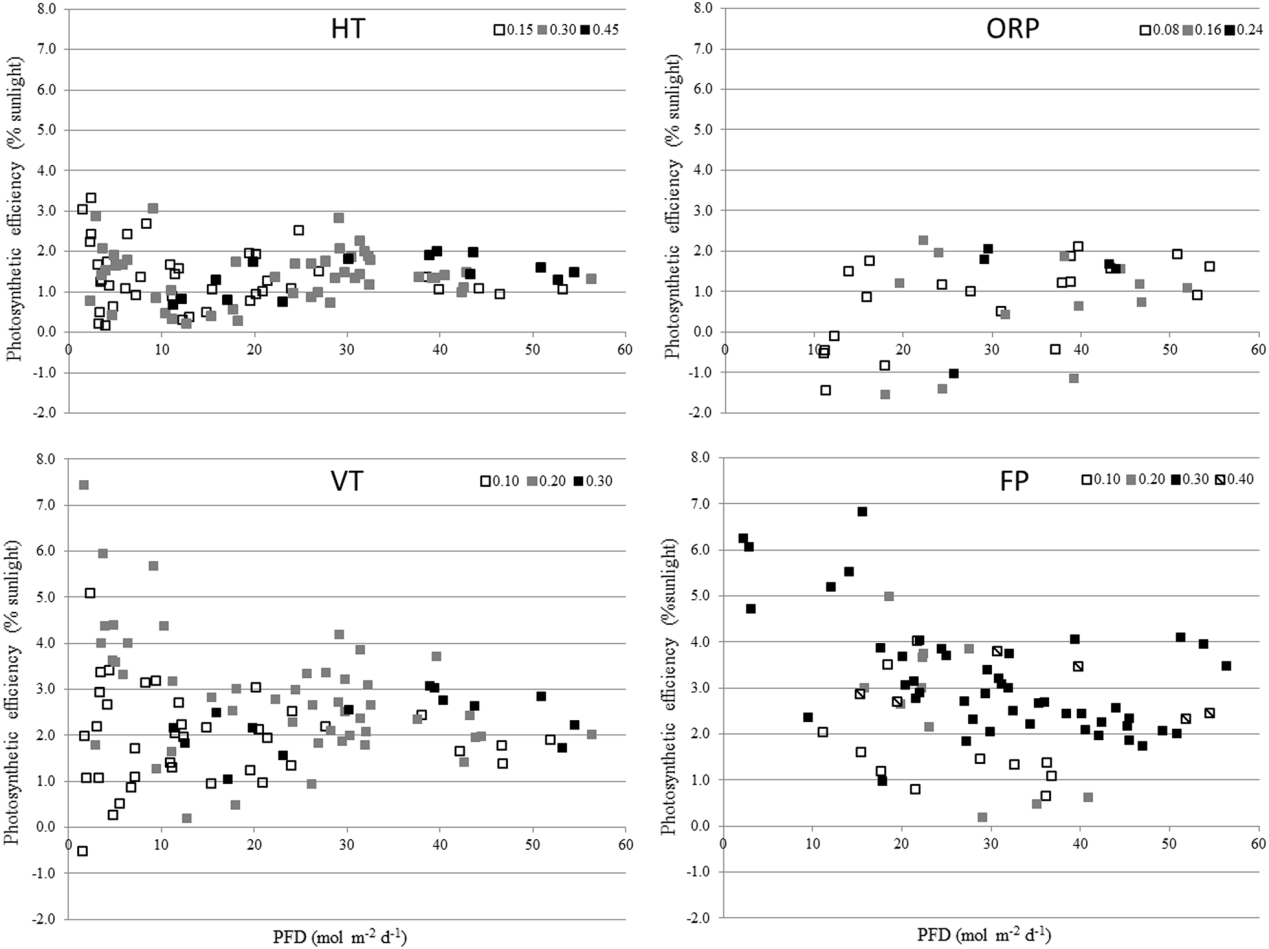
Influence of daily photon flux density on photosynthetic efficiencies on sunlight for the different photobioreactors. For the horizontal tubular photobioreactor (HT), open raceway pond (ORP), vertical tubular photobioreactor (VT) and flat panel photobioreactor (FP). The *different colors* of the *squares* indicate different dilution rates applied to each different photobioreactor

In Fig. 3, photosynthetic efficiencies versus photon flux densities are shown for all cultivation systems operated. For all systems, photosynthetic efficiencies varied over the range of photon flux densities. Maximal photosynthetic efficiencies were obtained for the three closed photobioreactors below 20 mol m^−2^ day^−1^. Furthermore, the more stable culture temperatures in the closed photobioreactors could have contributed to the higher photosynthetic efficiencies. Highest photosynthetic efficiencies were obtained with the vertical photobioreactors with intermediate dilution rates; 0.2 day^−1^ for VT and 0.3 day^−1^ for FP. Lower photosynthetic efficiencies were obtained with the horizontal tubular photobioreactor and the raceway pond. The photosynthetic efficiency is low in the horizontal tubular compared to the other closed systems. Variations in photosynthetic efficiencies were the result of the variations in areal productivities that were discussed before.

### Evaluation of performance

For a comparison of the performance of the cultivation systems among each other and with literature average and maximal values, for areal productivity and photosynthetic efficiency, were calculated over summer (Table 1). Volumetric productivities were calculated as these are often reported in literature.

**Table 1:** Overview of average and maximal areal and volumetric productivities and average and maximal photosynthetic efficiencies obtained in summer 2013 (July–August)

Photobioreactor	ORP		HT		VT		FP	
	Avg	Max	Avg	Max	Avg	Max	Avg	Max
*P*_x,ground_ (g m^−2^ day^−1^)	9.7	14.0	12.1	15.7	19.4	24.4	20.5	27.5
*P*_x,vol_ (g L^−1^ day^−1^)	0.03	0.08	0.65	0.85	0.57	0.71	0.90	1.20
Dilution rate (day^−1^)	0.14	0.12	0.25	0.34	0.27	0.40	0.27	0.36
Number of days	24	8	36	8	36	8	36	4
Photosynthetic efficiency (% sunlight)	1.1	1.5	1.5	1.8	2.4	4.2	2.7	3.8
Dilution rate (day^−1^)	0.16	0.12	0.25	0.28	0.27	0.24	0.27	0.18
Number of days	24	8	36	6	36	9	36	3

Dilution rates are measured values. Maximal areal and volumetric productivities were obtained in a single week in July with a high average photon flux density; 44 mol m^−2^ day^−1^

The highest average areal productivity was found in the flat panel photobioreactor, followed by the vertical tubular, the horizontal tubular photobioreactor and open raceway pond. Maximal areal productivities for each photobioreactor were obtained in a single week in July with a high average daily photon flux density of 44 mol m^−2^ day^−1^. The highest average photosynthetic efficiency was found for the flat panel (FP) photobioreactor followed by the vertical tubular (VT) photobioreactor, horizontal tubular (HT) photobioreactor and the open raceway pond (ORP). The highest maximal photosynthetic efficiency was found for the VT; followed by the FP, HT and ORP.

In the flat panel photobioreactor the highest areal and volumetric productivities and photosynthetic efficiencies were obtained. The highest volumetric productivity obtained for the FP (1.20 g L^−1^ day^−1^) is higher than values reported in literature, with the exception of data reported by Zou et al., of 1.7 g L^−1^ day^−1^ [5] (Table 2). However, this higher volumetric productivity was obtained in a flat panel photobioreactor with a shorter optical path of 1.3 cm, resulting in a higher light supply per volume of culture [5]. The photosynthetic efficiency obtained in this study for the flat panel photobioreactor is almost double of the values reported by Camacho-Rodriguez et al. for *Nannochloropsis gaditana* (1.7–0.3 %) and Rodolfi et al. for *Nannochloropsis* sp. F&M-M24 (0.96 %) [15, 16].

**Table 2:** Overview of volumetric and areal productivities and photosynthetic efficiencies (PE_sunlight_) for different photobioreactors outdoors reported in literature

Photobioreactor	Optical path (cm)	Algal species	*P*_x,vol_ (g L^−1^ day^−1^)	*P*_x,ground_ (g m^−2^ day^−1^)	PE_sunlight_ (%)	Author	Location
Horizontal tubular	4.3	*Nannochloropsis* sp.	0.51–0.76	13–19.5^a^	2.3–3.5^a^	[19]	Italy
Horizontal tubular	9.0	*Nannochloropsis gaditana*	0.12–0.20	10.8–18.0	0.7–1.04^a^	[15]	Almeria Spain
Horizontal tubular	4.6	*Nannochloropsis* sp.	0.30–0.85	5.8–15.7	1.2–1.8	This study	The Netherlands
Vertical panel	1.2	*Nannochloropsis* sp.	0.61–1.45	5.8–10.2	2.0–3.5^a^	[17]	Artificial light
Vertical tubular	10.4	*Scenedesmus obliquus*	?	21.76	2.5^a^	[20]	South Spain
Vertical tubular	5	*Nannochloropsis gaditana*	0.59	15.4	–	[18]	Almeria Spain
Vertical tubular	4.6	*Nannochloropsis* sp.	0.31–0.71	10.6–24.4	2.4–4.2	This study	The Netherlands
Raceway pond	30	*Scenedesmus obliquus*	0.03	8.26	0.95^a^	[20]	South Spain
Raceway pond	30	*Muriellopsis* sp.	0.04	8–20	0.97–0.69^a^	[21]	South Spain
Raceway pond	12	*Nannochloropsis salina*	0.2	24.5	–	[23]	Israel
Raceway pond	11	*Nannochloropsis gaditana*	0.09–0.19	22.4	–	[22]	Almeria Spain
Raceway pond	20	*Nannochloropsis* sp.	0.03–0.08	6.2–14.0	0.5–1.5	This study	The Netherlands
Flat panel	1.3–17	*Nannochloropsis* sp.	1.7–0.25	11–22	–	[5]	Israel
Flat panel	10	*Nannochloropsis* sp.	0.27	14.2	–	[28]	Israel
Flat panel	5	*Nannochloropsis gaditana*	0.16–0.36	8–18	1.74–0.31^a^	[15]	Almeria Spain
Flat panel	4.5	*Nannochloropsis* sp.	0.36	15.8	0.96^a^	[16]	Italy
Flat panel	5	*Nannochloropsis oculata*	0.15–0.37		–	[29]	Colorado, USA
Flat panel	2	*Nannochloropsis* sp.	0.9–1.2	20.5–27.5	2.7–3.8	This study	The Netherlands

The values for the raceway pond and FP for this study were collected in summer 2013. For both tubular photobioreactors average productivities and photosynthetic efficiencies were used to indicate the range of productivities and photosynthetic efficiencies; average data were obtained over the period from July to December 2013

^a^Calculated based on the illuminated area, not considering the ground area occupied by the photobioreactor

In the vertical tubular photobioreactor similar photosynthetic efficiencies (2–3.5 %) were obtained as for a modular flat panel system illuminated with artificial light as reported by Zittelli et al., [17]. In our study a lower volumetric productivity (0.3–0.7 g L^−1^ day^−1^) was obtained than values reported by Zittelli et al. [17] because of a larger optical path; 0.05 vs 0.012 m. The larger optical path could result in the formation of a dark zone in our system; resulting in a lower volumetric productivity. In our study a higher areal productivity (24 g m^−2^ day^−1^) was obtained than the areal productivity reported by Zittelli et al. (10 g m^−2^ day^−1^). Higher photon flux density than the photon flux density used by Zittelli et al., was measured outdoors, which contributed to the higher areal productivity obtained in our study. San Pedro et al., reported a maximal areal productivity of 15 g m^−2^ day^−1^ or 0.59 g L^−1^ day^−1^ for *Nannochloropsis gaditana* at a dilution rate of 0.3 per day [18]. These values are in the range of the values obtained in this study.

Volumetric productivities (0.3–0.85 g L^−1^ day^−1^) for the horizontal tubular photobioreactor obtained in this study are similar to the volumetric productivities (0.5–0.7 g L^−1^ day^−1^) reported by Zittelli et al. [19]. Lower volumetric productivities (0.12–0.2 g L^−1^ day^−1^) were reported by Camacho-Rodriguez et al., probably due to the larger tube diameter (9 cm). In our design, distance between tubes equals the diameter of the tube (external diameter 5 cm), this results in a lower culture volume per ground area, resulting in lower areal productivities than values reported by Zittelli et al. [19]. Camacho-Rodriguez et al. found similar areal productivities (10–18 g m^−2^ day^−1^) as in this study for *Nannochloropsis gaditana* cultivated in a horizontal tubular photobioreactor [15].

Areal productivities obtained for the open raceway pond in this study, 6–14 g m^−2^ day^−1^, were lower than the areal productivities reported by Arbib et al. and Blanco et al. (8–20 g m^−2^ day^−1^), due to the higher photon flux densities at the locations of the studies of Arbib et al. and Blanco et al. [20, 21]. Higher photon flux densities penetrate further and reduce the dark zone in a culture. Furthermore, in the south of Spain higher ambient temperatures are present, avoiding low culture temperatures down to 15 °C at night as experienced in our study. San Pedro et al., found maximal volumetric and areal productivity (0.19 g L^−1^ day^−1^ and 22.4 g m^−2^ day^−1^) for shallow (11 cm deep) raceway ponds [22]; the lower depth results in a smaller dark zone. In the study of San Pedro, higher productivities were obtained at higher photon flux densities and at temperatures close to optimum for growth [22]. Boussiba et al. reported higher areal productivity (24.5 g m^−2^ day^−1^) as well for *Nannochloropsis salina* cultivated in a shallow pond [23], this indicates that lower culture depth or more light per culture volume results in higher productivity.

## Conclusions

The performance of different pilot-scale photobioreactor designs under identical conditions was evaluated. Flat panel photobioreactors resulted in high ground areal productivities (≥24 g m^−2^ day^−1^) and high ground areal photosynthetic efficiencies (≥2.7 %) over 36 days. Average photosynthetic efficiencies for the other systems were: VT; 2.4 %, HT; 1.5 % and ORP; 1.2 %.

Vertical photobioreactors resulted in higher areal productivities than horizontal photobioreactors because of the higher light interception and the resulting lower incident photon flux densities on the reactor surface. Among the vertical photobioreactors studied, the flat panel photobioreactor showed the highest average photosynthetic efficiency, areal and volumetric productivities due to its short optical path.

Concluding, photobioreactor light interception should be optimized to maximize ground areal productivity and photosynthetic efficiency. This makes vertical photobioreactors promising for large scale production. However, an economical analysis should be made to assess if the higher photosynthetic efficiency and higher areal productivity compensate for the higher investment costs generally associated with vertical photobioreactors.

## Methods

### Inoculum production

*Nannochloropsis* sp. CCAP 211/78 was cultivated in enriched natural seawater (Oosterschelde, The Netherlands) with the following concentrations (in mM); NaNO_3_, 25; KH_2_PO_4_, 1.7; Na_2_EDTA, 0.56; Fe_2_SO_4_·7H_2_O, 0.11; MnCl_2_·2H_2_O, 0.01; ZnSO_4_·7H_2_O, 2.3·10^−3^; Co(NO_3_)_2_·6H_2_O, 0.24·10^−3^; CuSO_4_·5H_2_O, 0.1·10^−3^; Na_2_MoO_4_·2H_2_O, 1.1·10^−3^. For the pre-cultures (250 mL Erlenmeyer flasks) and cultivation in the 4.5 L flat panel reactor, HEPES (20 mM) and Na_2_EDTA (5 mM) were added to the seawater. The pH was adjusted to 7.5 followed by sterilization (121 °C, 20 min); after sterilization, nutrients were added to the sterilized seawater through a sterile filter (0.45 µm). For all other cultivations (including outdoor cultivations), seawater was chemically sterilized (sodium hypochlorite), active chlorite was deactivated by filtration over active carbon, followed by filtration (1 μm).

The pre-cultures were placed in an orbital shaker incubator (Multitron, Infors HT, the Netherlands). Cultures were shaken at 120 rpm, illuminated with 50 µmol m^−2^ s^−1^, at a temperature of 25 °C and headspace was enriched with 2 % CO_2_. The Erlenmeyer flasks were used as inoculum for cultivation in a 4.5 L flat panel photobioreactor (optical path 2.5 cm); pH was controlled at 7.5 by on demand CO_2_ addition, temperature was controlled at 25 °C and mixing by aeration at 1.5 L^−1^ L^−1^ min^−1^. The harvest of this 4.5 L reactor was used to inoculate a 280 L horizontal tubular photobioreactor placed in a greenhouse. Temperature was maintained at 25 °C, pH was controlled at 7.5 by on demand CO_2_ addition. This photobioreactor was operated at a liquid velocity of 0.3 m s^−1^. To increase production, six 600 W high-pressure sodium lamps (Master SON-T PIA Green Power, Philips Eindhoven, The Netherlands) were placed above the transparent tubular section of the reactor, which in addition to sunlight delivered a photon flux density of 350 µmol m^−2^ s^−1^. All outdoor photobioreactors were inoculated within one week with the harvest from this system.

### Outdoor pilot-scale photobioreactors

**Fig. 4:**
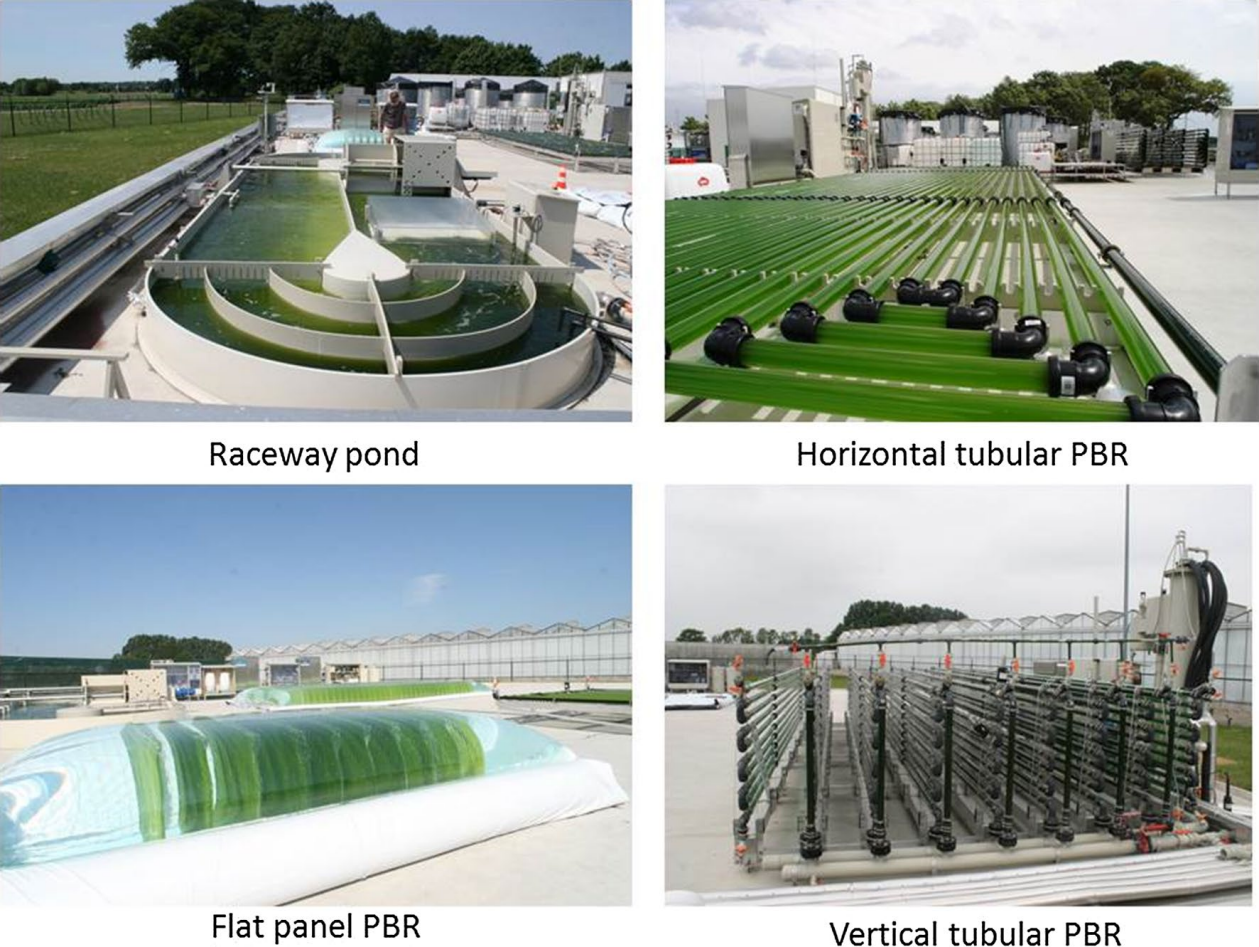
Photobioreactors in operation at AlgaePARC pilot facilities, Wageningen UR, the Netherlands

A short description of each photobioreactor (Fig. 4) is given in this section; a more detailed description of the outdoor systems is given by Bosma et al. [6]. All cultivation systems were operated at a pH of ±7.5 by on demand CO_2_ addition and culture temperatures were maintained between 20 and 30 °C. Specifications of the different photobioreactors studied are given in Table 3.

**Table 3:** Specifications of outdoor pilot-scale photobioreactors

Specifications	ORP	HT	VT	FP
Optical path (m)	0.2	0.046	0.046	0.02^a^
Volume (m^3^)	4.73	0.56	1.06	0.06
Illuminated volume (%)	100	73	71	100
Ground area occupied (m^2^)	25.4	27.0^b^	31.0^b^	2.4
Illuminated volume/ground area (m^3^ m^−2^)	0.186	0.021	0.034	0.023
Expected PE_sunlight_ (%)^c^	1.5	3	n.a.	5

*ORP* open raceway pond, *HT* horizontal tubular, *VT* vertical tubular, *FP* flat panel photobioreactor

^a^Average optical path of single panels

^b^Including half of ground area occupied by dummy panels/tube installed at northern and southern side of reactors

^c^Data from Norsker et al. [3]

### Open raceway pond

The raceway pond has an optical path of 0.2 m and water was circulated in the pond using a paddle wheel (L 1.45 m, W 0.235 m). The liquid velocity in the OPR was 0.25 m s^−1^. Carbon dioxide was injected on demand for pH control. At the point of injection a transparent cover was built above culture level to recirculate the gas phase and prevent excessive carbon dioxide losses. There was no need for active oxygen removal in the open raceway pond, as dissolved oxygen concentrations never reached values above 160 %.

### Horizontal tubular photobioreactor

The horizontal tubular photobioreactor consists of three loops of 80 m each (Fig. 4) connected via a manifold to a bubble column used for oxygen removal, temperature control, nutrient and antifoam addition (Silicone RE20 Snapsil, BRB international, The Netherlands). The horizontal photobioreactor was operated at a liquid velocity of 0.45 m s^−1^. To prevent high concentrations of dissolved oxygen, a superficial gas velocity (*v*_gs_) of 0.04 m s^−1^ was used in the bubble column (volume of 0.15 m^3^, 27 % of total reactor volume). High dissolved oxygen concentrations above 300 % hamper the growth of *Nannochloropsis* sp. under dynamic oxygen concentrations [23]. Similar dissolved oxygen concentrations were found inhibiting for *Neochloris oleoabundans* [24]. The airflow in the bubble column was increased when dissolved oxygen concentration exceeded 300 % to prevent inhibiting oxygen concentrations.

### Vertical tubular photobioreactor

The vertical tubular photobioreactor consists of seven vertical loops of 80 m each connected by a manifold to a bubble column used for oxygen removal, temperature control, nutrient and antifoam addition. The liquid velocity in the tubes was 0.45 m s^−1^. To prevent high concentrations of dissolved oxygen, a superficial gas velocity of (*v*_gs_,) 0.04 m s^−1^ air was used in the bubble column (volume 0.31 m^3^, 29 % of total reactor volume). As in the horizontal system, the superficial gas velocity was increased when dissolved oxygen concentration exceeded 300 % to decrease the dissolved oxygen concentration in the culture. On the northern and southern side of the reactor a dummy panel, filled with a green dye, was placed to prevent that the first and last panel receive a lot of direct light.

### Flat panel photobioreactor

The flat panel system consists of 10 vertical panels (width 1.25 m, height 0.5 m, depth 0.02 m), with 0.25 m distance between the panels. The total occupied ground surface was 2.4 m^2^ and thus 10 times smaller than that of the other photobioreactors (Table 3) studied. In contrast to the other systems, the culture was not mixed over the entire reactor; passive mixing takes place over a panel. In addition, the culture moves in a plug flow manner through each panel; fresh media is added at one side of the panel and simultaneously harvesting is done at the other side by overflow. The culture in the flat panels was mixed by gassing the culture at a rate of 1 L^−1^ L^−1^ min^−1^ (*v*_gs_ 0.01 m s^−1^). The gas phase was continuously recycled and the carbon dioxide and oxygen concentration in this gas phase were continuously monitored. pH control was achieved by addition of pure carbon dioxide whenever the concentration decreased below 1 % v/v. High dissolved oxygen concentrations were prevented by bleeding a part of the recirculated gas flow as soon as the oxygen concentration in the gas phase exceeded 30 % v/v. The large water volume surrounding the panels acts as a temperature buffer and can be cooled or heated via a heat exchanger (Fig. 4).

### Harvesting regime

The photobioreactors were diluted with a fixed daily dilution rate for 7 days. After 7 days, dilution rate was changed to the next dilution rate (Table 4). The range of dilution rates for each photobioreactor was set based on growth rates determined in these systems in 2013 (unpublished data). In the tubular and flat panel photobioreactors dilution rates were applied for each experimental run in the following order; medium, low, medium, high, medium. In the raceway pond the short operational timeframe did not allow the repetition of the intermediate dilution rate. The culture in tubular systems and raceway pond were diluted by harvesting several small volumes distributed over the day from the reactor (every hour for 15 min between 10:00 and 15:00) and adding sterilized natural seawater during daytime and nutrients. In the raceway pond, nutrients were added flow proportionally to the flow of seawater with a Dosmatic Minidos 12 system. The flat panel was harvested once at 9:00 a.m. and diluted with complete medium (nutrient stock enriched seawater) that was prepared in a separate vessel.

**Table 4:** Overview of the four dilution rates (day^−1^) applied to each photobioreactor

Photobioreactor/dilution rate	*D*1	*D*2	*D*3	*D*4
Open raceway pond	0.08	0.16	0.24	
Horizontal tubular	0.15	0.30	0.45	
Vertical tubular	0.10	0.20	0.30	
Flat panel reactor	0.10	0.20	0.30	0.40

### Measurements and analysis

The photobioreactors were sampled daily between 9:00 and 10:00 a.m. for optical density measurement (680 and 750 nm) on a DR5000 spectrophotometer (Hach Lange, Germany). From the same samples, three times a week dry weight determinations were done in triplicate as described by Zhu et al. [25]. The dry weight concentration was correlated to the optical density measured at 750 nm (OD_750_). The harvested volume was determined daily for each photobioreactor and the harvest was mixed by a pump and then sampled for optical density measurement at 680 nm (OD_680_) and at 750 nm (OD_750_). The OD_750_ measurements of the harvest and of sample taken from the reactors were used to calculate the productivity of each photobioreactor. Nitrate concentrations in the harvest were maintained above 1 mM to ensure nutrient replete conditions. For this, the nitrate content of a sample from the harvest vessel was measured; 2 mL was centrifuged and the supernatant was analysed for nitrate content with an AQ-2 nutrient analyser, (Seal Analytic, USA) as described by Benvenuti et al. [26] (HMSO, 1981; APHA/AWWA/WEF, 4500; USEPA, 19932).

### Calculations

All values were calculated over a period between two consecutive sampling points with Eqs. 1–3.

### Ground areal biomass productivity

**Fig. 5:**
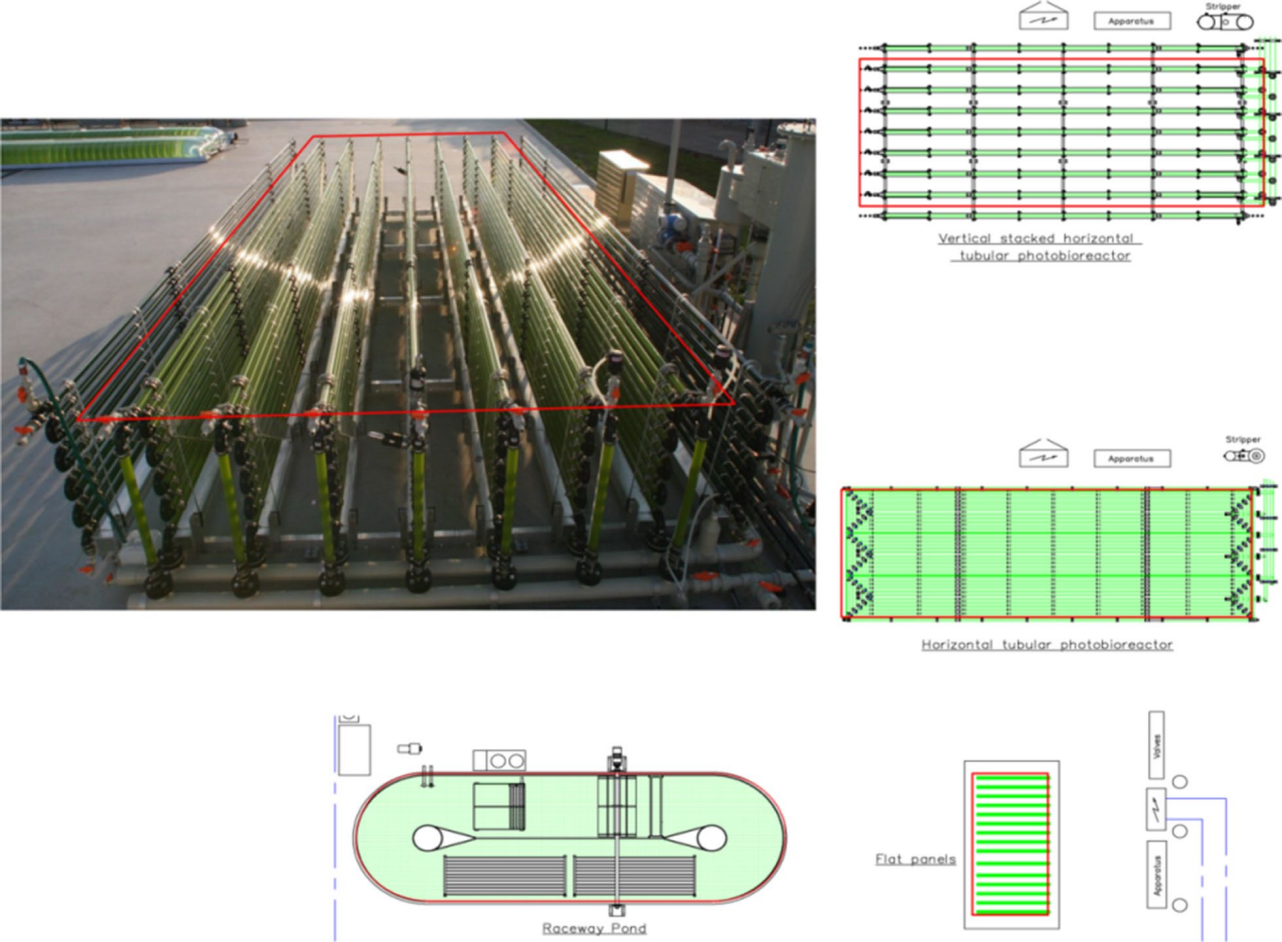
Ground area considered for calculation of areal productivity and photosynthetic efficiency for each photobioreactors. *Top left* photograph of vertical tubular photobioreactor with area considered as ground area indicated by *red lines*

Daily ground areal biomass productivities were calculated with Eq. 1. In Eq. 1 the accumulation of biomass in the reactor and the harvested biomass were taken in account.

1 $$P_{\text{x,ground}} = \left( {\frac{{(V_{\text{harvest}} \times C_{\text{x,harvest}} ) + (V_{\text{R}} \times C_{\text{x}} (t) - C_{\text{x}} (t - 1))}}{{A_{\text{ground}} }}} \right){\text{g m}}^{ - 2} {\text{day}}^{ - 1}$$

with: *P*_x,ground_: ground areal biomass productivity (g m^−2^ day^−1^); *V*_harvest_: harvested volume (L); *C*_x, harvest_: dry weight algal concentration in the harvest (g L^−1^); *V*_R_: photobioreactor volume (L); *A*_ground_: occupied ground area photobioreactor (m^2^); *C*_x_(*t*), *C*_x_(0): dry weight algal concentration in photobioreactor (g L^−1^), on consecutive sampling points; *t*: time between consecutive sampling points; ±24 h. For the calculation of the ground area, the area used is indicated in Fig. 5.

In the tubular systems a dummy tube in HT and in VT a dummy panel was placed on the northern and southern side. The dummy tubes and dummy panels were filled with green dye to exclude the side panels/tube of receiving more direct light. The installation of dummy panels in the flat panel PBR was not possible. For the calculation of the ground area in the flat panel, the area taken up by the panels was considered and not the area of the entire bag as this area would be smaller in a larger version of the photobioreactor.

### Volumetric biomass productivity

The volumetric biomass productivity was calculated from the ground areal productivity with Eq. 2.

2 $$P_{\text{x,vol}} = P_{\text{x,ground}} \times \frac{{A_{\text{ground}} }}{{V_{\text{R}} }}{\text{g L}}^{ - 1} {\text{day}}^{ - 1}$$

with: *P*_x,vol_: volumetric productivity (g L^−1^ day^−1^); *P*_x,ground_: ground areal biomass productivity (g m^−2^ day^−1^); *V*_R_: photobioreactor volume (L); *A*_ground_: ground area photobioreactor (m^2^).

### Photon flux density

3 $$I_{\text{ground,daily}} = \sum\limits_{t = 1}^{t = 1440} {I_{\text{ground}} (t) \times 60} \times 10^{ - 6} {\text{mol m}}^{ - 2} {\text{day}}^{ - 1}$$

The daily ground areal photon flux density (*I*_ground,daily_ mol m^−2^ day^−1^) was calculated with Eq. 3. Ground areal photon flux densities (*I*_ground_; µmol m^−2^ s^−1^) were measured on a horizontal plane every minute with a Li-Cor-190SA 2*π* PAR quantum sensor (LiCOR, USA) present at the AlgaePARC pilot facility. The photon flux densities measured every minute, between two consecutive reactor sampling points (±24 h), were summed and multiplied by 60 (i.e., conversion from seconds to minutes).

### Photosynthetic efficiency on sunlight

The photosynthetic efficiency was calculated with Eq. 4.

4 $$PE_{\text{sunlight}} = \frac{{(P_{\text{x,ground}} \times \varDelta H_{c}^{o} )}}{{((I_{\text{ground,daily}} \times (0.43 \times E_{\text{PAR}} ))/10^{3} )}}$$

with: *PE*_sunlight_: photosynthetic efficiency (% sunlight); *P*_x,ground_: average ground areal productivity (g m^−2^ day^−1^); $\varDelta H_{c}^{o}$: standard enthalpy of combustion (22.5 kJ g^−1^); *I*_ground,daily_: average daily areal photon flux density, Eq. 3 [mol m^−2^ day^−1^ (PAR, photosynthetic active radiation)]; *E*_PAR_: energetic content of the PAR fraction of sunlight (4.76 J mol^−1^), ASTM G173-03 [27]; and 0.43 the conversion factor from sunlight to PAR light on an energy basis (J J^−1^).

## Nomenclature

### List of symbols

PE_sunlight_: sunlight to biomass conversion efficiency (%)
*P*_x,ground_: ground areal biomass productivity (g m^−2^ day^−1^)
*V*_harvest_: harvested volume (L)
*C*_x_: biomass concentration (g L^−1^)
*V*_r_: volume of photobioreactor (L)
*A*_ground_: ground area occupied by photobioreactor (m^2^)
*P*_x,vol_: volumetric biomass productivity (g L^−1^ day^−1^)
*I*_ground,daily_: daily ground areal photon flux density (mol m^−2^ day^−1^)
*I*_ground_: ground areal photon flux density (µmol m^−2^ s^−1^)
$$\varDelta H_{c}^{o}$$
standard enthalpy of combustion (kJ g^−1^)
*E*_PAR_: conversion factor PAR photons to joule (J mol^−1^)
*D*: dilution rate (day^−1^)
OP: optical path (cm)
PFD: photon flux density (mol m^−2^ day^−1^)
v_gs_: superficial gas velocity (m s^−1^)

### Abbreviations

ORP: open raceway pond
HT: horizontal tubular
VT: vertical tubular
FP: flat panel
